# Dr. D. Meredith Reese

**Published:** 1861-07

**Authors:** 


Dr. D. Meredith Reese died at New York on the thirteenth of May, of disease of the heart. At the time of his death he was Professor of Medicine in the Medical College of New York, and Editor of the *American Medical Gazette*, which he founded about ten years ago.

We extract, from an obituary notice in the *New York Medical Times*, the following particulars of Dr. Reese's life:—

"Dr. Reese was born in Maryland, about the year 1800. He graduated in medicine at the University of Maryland, March 26, 1819, his inaugural thesis being De Mania Religiosa, and settled in practice at Baltimore. He passed through the epidemic fever which devastated that city in 1819, and wrote a 12mo. pamphlet upon it soon after its disappearance. He was afterward appointed 'Professor of the Institutes of Medicine and Surgery and Medical Jurisprudence in the Washington University of Baltimore,' and subsequently held professorships in the Albany Medical College, New York, and the Castleton Medical College, Vermont. It was about this time that Dr. Reese took up his residence in New York. He acquired so much professional and political influence as to be appointed Resident Physician at Bellevue Hospital, a position which he retained for several years, until the office was abolished in 1849.

"Dr. Reese was one of the original members of the Academy of Medicine, and drafted its first constitution. He always took a deep interest in its affairs, was seldom absent from its meetings, and entered heartily into its discussions. He was a ready and fluent speaker, a good debater, familiar with parliamentary rules, and often succeeded in carrying his point by the skillful use of this knowledge. At the meeting of the American Medical Association held at Nashville, Tenn., in 1857, Dr. Reese was elected second Vice-President.

"As a writer, Dr. Reese was widely known, not only in medicine, but in politics, religion, etc. He wielded a rapid and vigorous pen; but he was neither happy in the choice of his subjects, nor in the manner of treating them. His arguments were too often specious, his style inflated, and his illustrations inapt. He exhibited an almost total want of power of discriminating the true character and motives of men, and was thus frequently led to attack with great vehemence the best members of the profession, and attribute to them motives which a generous mind would scorn to entertain. This unfortunate peculiarity brought him in constant and unfriendly collision with his professional brethren, and completely nullified his influence. His most useful papers were his reports to the American Medical Association, the last of which, on Medical Education, is replete with mature and well-digested views of this all-important subject."

